# “Having surgery is necessary” – a qualitative analysis of the experiences of frail older adults treated with, and recovering from colorectal cancer surgery

**DOI:** 10.1186/s12877-026-07356-3

**Published:** 2026-03-17

**Authors:** Maria Normann, Niklas Ekerstad, Mattias Prytz, Erika Björklund, Kristina Åhlund

**Affiliations:** 1 Department of Surgery, Institute of Clinical Sciences, Sahlgrenska Academy, University of Gothenburg, Gothenburg, Sweden; 2 Department of Surgery, Region Västra Götaland, NU-Hospital Group, Trollhättan, Sweden; 3 Department of Health, Medicine and Caring Sciences, Linköping University, Linköping, Sweden; 4 Department of Research and Development, Region Västra Götaland, NU-Hospital Group, Trollhättan, Sweden; 5 Department of Health Sciences, University West, Trollhättan, Sweden; 6 Department of Orthopaedics, Institute of Clinical Science, Sahlgrenska Academy, University of Gothenburg, Gothenburg, Sweden

**Keywords:** Colorectal cancer, Oncological Surgery, Frailty, Older adults, Interview study

## Abstract

**Background:**

Frail older adults undergoing surgical treatment for colorectal cancer are at increased risk of postoperative mortality and complications compared with non-frail older adults. Even though these risks are established, limited research has explored patient perspectives on surgical treatment and recovery in frail older adults. This study aimed to explore the experiences of frail older adults during diagnosis, treatment, and recovery from surgically treated colorectal cancer.

**Methods:**

Participants were recruited through purposive sampling from the control group of the randomized controlled trial “*Effect of comprehensive geriatric assessment for frail elderly patients operated for colorectal cancer – the Colorectal Cancer Frailty Study*.” Patients in the control group receive standardised treatment according to best practice within an ERAS-concept. Sixteen semi-structured interviews were conducted 6–22 months post-surgery. All interviews were audio-recorded, transcribed verbatim, and analysed using qualitative content analysis with a focus on both manifest and latent content.

**Results:**

Participants perceived surgery as essential for survival. They expressed considerable trust in health care professionals and showed limited interest in being actively involved in medical decision-making. Even among those who experienced postoperative challenges—such as reduced physical function and increased dependence on others—there was a high level of acceptance and a prevailing sense that the treatment had been worthwhile. Overall, the experience of the treatment was described in a positive sense, and participants stated that they would choose to undergo the procedure again if necessary.

**Conclusion:**

The findings of this study revealed that participants sought clear and concrete recommendations and expressed limited interest in influencing medical decision-making. There was a strong will to undergo surgery despite complicating factors such as advanced age, frailty, and comorbidities. A general acceptance of age-related functional decline may have contributed to the sense of being content with treatment outcomes, even in the presence of postoperative challenges. The findings reflect the perspective of surgically treated, mild to moderately frail older adults with colorectal cancer, who survived at least six months after surgery, and the results should be interpreted within this context.

**Trial registration:**

OSF registry: https://osf.io/c94xb/, registered May 24, 2025.

**Supplementary Information:**

The online version contains supplementary material available at 10.1186/s12877-026-07356-3.

## Background

Colorectal cancer is the third most common cancer globally, most new cases are amongst people ≥ 70 years, and the incidence is increasing [1–3]. Frailty is a condition of reduced reserves and increased vulnerability. It is associated with advanced age, morbidity and disability and can be used as a marker of biological age [4, 5]. It is known that elderly patients have higher postoperative mortality compared to younger individuals, and the tendency is even clearer in frail older adults [6–8]. Our research group have previously published work on post-operative mortality, complications and length of stay following colorectal cancer surgery in elderly and in frail elderly individuals in a Swedish setting. These studies confirm that frail patients suffer increased risks of post-operative mortality both in short- and long-term compared to non-frail patients of the same age [9, 10]. Due to the demographic evolvement in the Western world, with increased life expectancy and readily access to healthcare, one can only assume that colorectal cancer surgery in older adults will continue to increase, and efforts should be made to improve decision making and postoperative outcome [11].

It is not entirely understood how frail patients recover after colorectal cancer surgery, or how the treatment affects quality of life (QoL) and physical function. Previous studies have shown that older patients generally experience poorer health-related quality of life (HRQoL) and an accelerated functional decline after surgery, compared to younger patients [12, 13]. Regarding colorectal cancer surgery, one study described decreased physical function in adults aged > 70, but without association to frailty [14]. Further, studies exploring QoL in frail or functionally dependent patients, treated with surgery for colorectal cancer, showed an increase in QoL in both groups at follow-up, both in short- and long-term [15, 16]. Even though there is substantial knowledge regarding post-operative outcomes in terms of mortality, complications, and health care costs, less is known about what aspects of disease and treatment are most important in this patient category [17, 18].There is an increasing will to adopt the concept of person-centred care (PCC) in modern healthcare systems, and Swedish authorities encourage healthcare workers to apply a person-centred approach when treating patients. There is a general will to increase the individual’s possibility to influence and to be involved in planning and decision-making [19]. There are several definitions of PCC, but they all include certain key factors such as: shared decision-making, striving towards individualised treatment, having a biopsychosocial approach and patient empowerment. A question encircling the essence of the concept is “what is important to you?” [20, 21]. Since the population is growing older and in larger need of advanced healthcare, it is important to understand what measures and treatments are truly important to the individual [17]. Qualitative studies enable researchers to deepen the understanding of patients’ experiences. This can be an important complement to what is already known about frail patients and colorectal cancer surgery when advising and guiding patients regarding treatment options. The aim of the study was to examine frail elderly patients’ experiences of being treated for colorectal cancer, and explore what aspects were important during the process, including the recovery period.

## Methods

The manuscript follows the guidelines for reporting qualitative research (COREQ) [22]. The study has been approved by the Swedish Ethical Authority, Dnr: 2024-07171-02.

### Design

This study is a descriptive, qualitative study based on semi-structured interviews which were analysed through inductive content analysis, exploring both manifest and latent content. The manifest analysis is kept close to the text, focusing on the evident message and displayed as citations and categories within the article. The latent content is an interpretation of the underlying message and is displayed as themes [23]. An interview guide was developed specifically for this study and used as a template for the interviews that took place 6–22 months post-surgery, focusing on the patients’ experiences of the time prior to surgery, the period surrounding the surgery and the post-operative period.

### Setting

Participants were obtained from the control group of the randomised controlled trial “Effect of comprehensive geriatric assessment for frail elderly patients operated for colorectal cancer – the colorectal cancer frailty study”, from here on referred to as “The CRC Frailty study”. The study is a randomised, controlled, multicentre trial. Patients in the control group receive evidence-based standard care (best practice), whereas patients in the intervention group receive assessment and treatment according to best practice and in addition a comprehensive geriatric assessment (CGA) and care concept. The details of the study have been described previously [24]. The CRC Frailty study was at the time of interviews, ongoing at three hospitals in Region Västra Götaland (VGR) in Sweden and approved by the Swedish Ethical Review Authority (Dnr: 2019–05340). The main study was registered at ClinicalTrials.gov (NCT04358328) and this study at the OSF registry (https://osf.io/c94xb/). Patients in the current study were recruited from the control group of patients included at two county hospitals and one university hospital, in total serving a catchment area of approximately 1.2 million habitants.

### Participants

Patients eligible for inclusion in the CRC Frailty study were recently diagnosed with a colorectal cancer where curatively intended surgery was deemed possible, were aged ≥ 65 years and without significant cognitive impairment or language limitations. All patients were considered frail according to the Clinical Frailty Scale (CFS-9), that is, having a score of 4–8. Exclusion criteria in the CRC Frailty study were: a palliative situation, estimated life expectancy of under six months (CFS score 9), inability to understand study information, urgent/emergent surgery or unwillingness to participate. Patients randomised to control group received standardised care according to best known practice, within an Enhanced Recovery After Surgery (ERAS) concept. No general alterations of the standardised ERAS-protocol were made depending on patient’s frailty status. This includes evaluation at a multidisciplinary team conference (MDT), preoperative evaluation by a colorectal surgeon and anaesthesiologist, if necessary, interdisciplinary referrals to other specialists as well as a visit with a stoma specialist, if the planned surgery includes formation of an ostomy. The only addition to standard protocol for these patients are study-specific questionnaires at inclusion and at follow-up (8 weeks and 1 year postoperatively) [24]. Individual recommendations regarding structured follow-up were issued at a post-operative MDT, based on the results from the pathological analysis of the specimen, in combination with patient’s overall status considering comorbidities, age, frailty etc. Not all patients are planned for radiologic follow-up, in general due to early tumour stage and/or because the patient was not deemed able to receive any further treatment in case of recurrence. This decision, together with its underlying rationale, was communicated to patients during the postoperative follow-up visit.

**Fig. 1:**
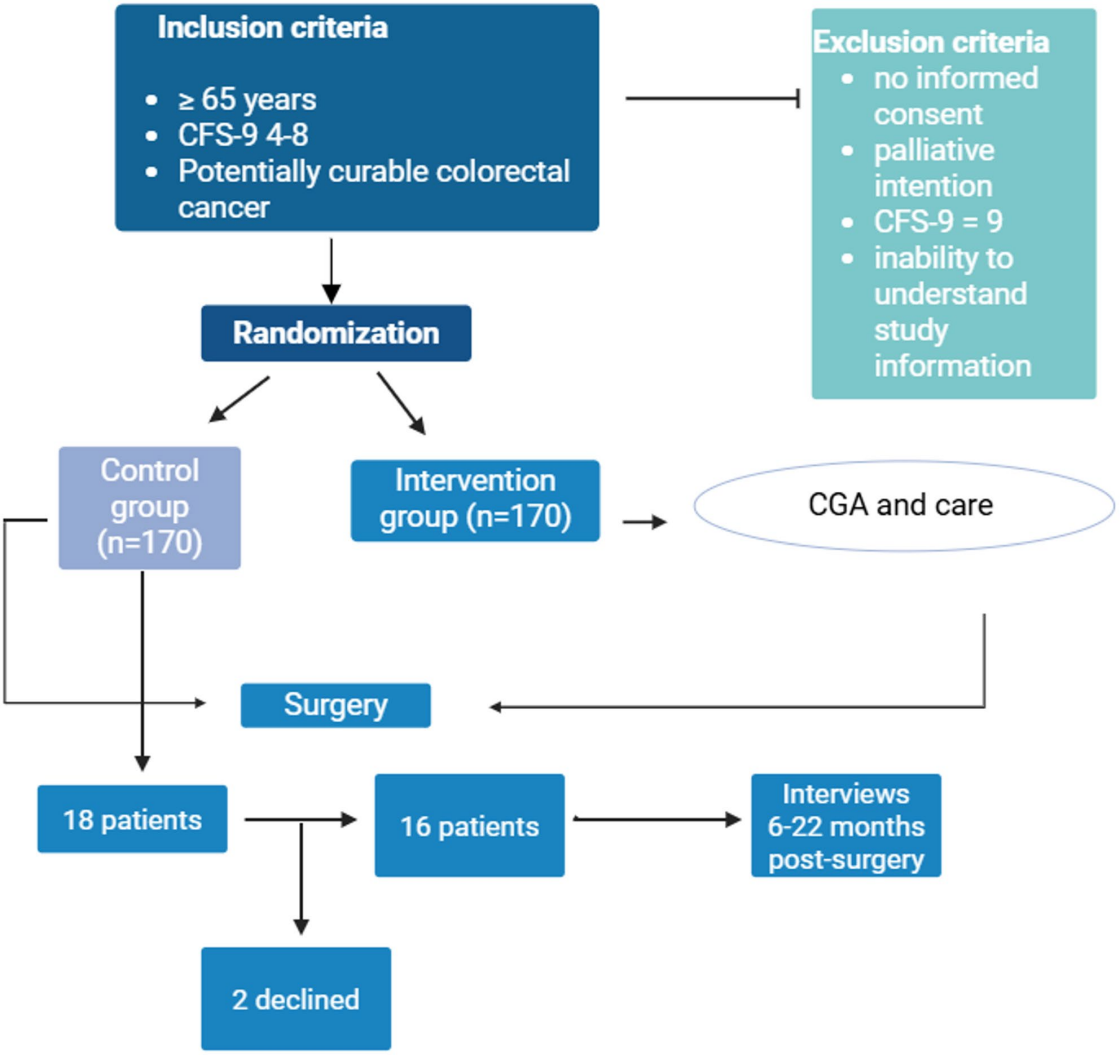
Flow chart describing the CRC Frailty study and the selection of patients in the current study

Purposeful sampling was used to select participants to the interview study, striving to include different experiences, to broaden the diversity and to deepen the understanding. Patients of different degrees of frailty, both men and women, of different ages, different housing situations, living in rural and urban areas, both colon and rectal cancer, with a broad time range of 6–22 months post-surgery, and patients who received adjuvant oncologic treatment and who did not, were recruited. The goal was to recruit 15–20 patients, in order to find repeating patterns as well as individual variations [23]. Eighteen patients were asked to participate in the study, of these two patients declined partaking, leaving a total number of 16 conducted interviews (Fig. 1). Data collection and analysis proceeded concurrently, with transcripts analysed iteratively to identify emerging findings. Interviews continued until no new themes emerged, at which point the sample was considered sufficient. Demographic characteristics of the study population are described in Table 1.

**Table 1:** Patient characteristics for all subjects

Variables		All subjects (*n* = 16)
Age (years)		83 (74–89)
Female		7 (44)
CFS-9		4 (4–6)
Living situation		
	*Living alone*	4 (25)
Tumour local		
	*Colon*	14 (88)
	*Rectum*	2 (12)
Permanent ostomy		7 (44)
Adjuvant oncologic treatment		2 (13)
Length of interview (min)		38 (23–58)
Time between surgery and interview (months)		12 (6–22)

The values were expressed as median (min-max) and number (%)

*Abbreviations*: *CFS-9*Clinical Frailty Scale 9

### Data collection

Patients were offered inclusion in the interview study after completion of the planned treatment, and the interviews took place 6–22 months after surgery. The time range was chosen to ensure perspectives of both recently received surgery and of more distant treatment, to ensure a broad perspective in relation to time of surgery. Eligible patients were approached by a letter with written information regarding the study, this was followed up with a phone call approximately one week later. Patients willing to participate were asked to sign a written consent. A meeting to conduct the interview was planned, and took place either in patients own home, or in a private room at the hospital, depending on patients own choice. In case the participant wanted a next of kin present during the interview, they were asked to limit their interaction during the conversation. In some cases, the presence of family members during the interviews was necessary to support participants in recalling details. When next of kin began to interfere with participants’ narratives, they were asked to step back and share their own reflections at the end of the interview, rather than intervening in the participants’ accounts.

A semi-structured interview guide was developed focusing on the patient’s experiences from diagnosis through treatment and the recovery period (supplementary file). The interview guide was developed within the research group, based on previous research experience and clinical expertise. The interview guide was not piloted or altered during data collections. The questions focused on how the treatment has affected their daily life, with the opening question “what were your thoughts when you were told that you were going to have surgery for your cancer?”. MN, KÅ and EB conducted the interviews in pairs, the interviews were audio recorded and later transcribed. The transcriptions were not returned to participants for correction after interviews. Clinical data regarding demographics and treatments was gathered from the participants medical charts.

### Data analysis

To limit subjectivity in the analysis the process followed the content analysis methodology [23]. Data analysis consisted of repeated reading of the interviews, focusing on grasping both manifest and latent content. The core content from the narratives were condensed into meaning units. These were organised into codes, which serves as labels describing the key concepts. Codes regarding similar topics were grouped in categories. Further abstractions of the latent meaning were used to create themes as a way of capturing the underlying meaning of the text. The interviews and subsequent analyses were conducted in Swedish. The relevant quotes were translated into English by the first author during manuscript preparation. The translations were discussed within the author group and, where necessary, altered to preserve nuance and meaning. As translations were conducted after data analysis, it was not considered to have influenced the study findings. The first analyses were independently made by MN (PhD-student, MD, surgeon), KÅ (PhD, physiotherapist) and EB (registered nurse) and then compared. If different conclusions were made, results were discussed until consensus was reached using an iterative process. These first analyses were presented and discussed with MP (associate professor, MD, colorectal surgeon) and NE (associate professor, MD, cardiologist). Patients eligible for inclusion in this study had already received their planned treatment and none of the persons who conducted the interviews had previously been responsible in the care or treatment of the participant in question, nor in their planned follow-up.

## Results

**Fig. 2:**
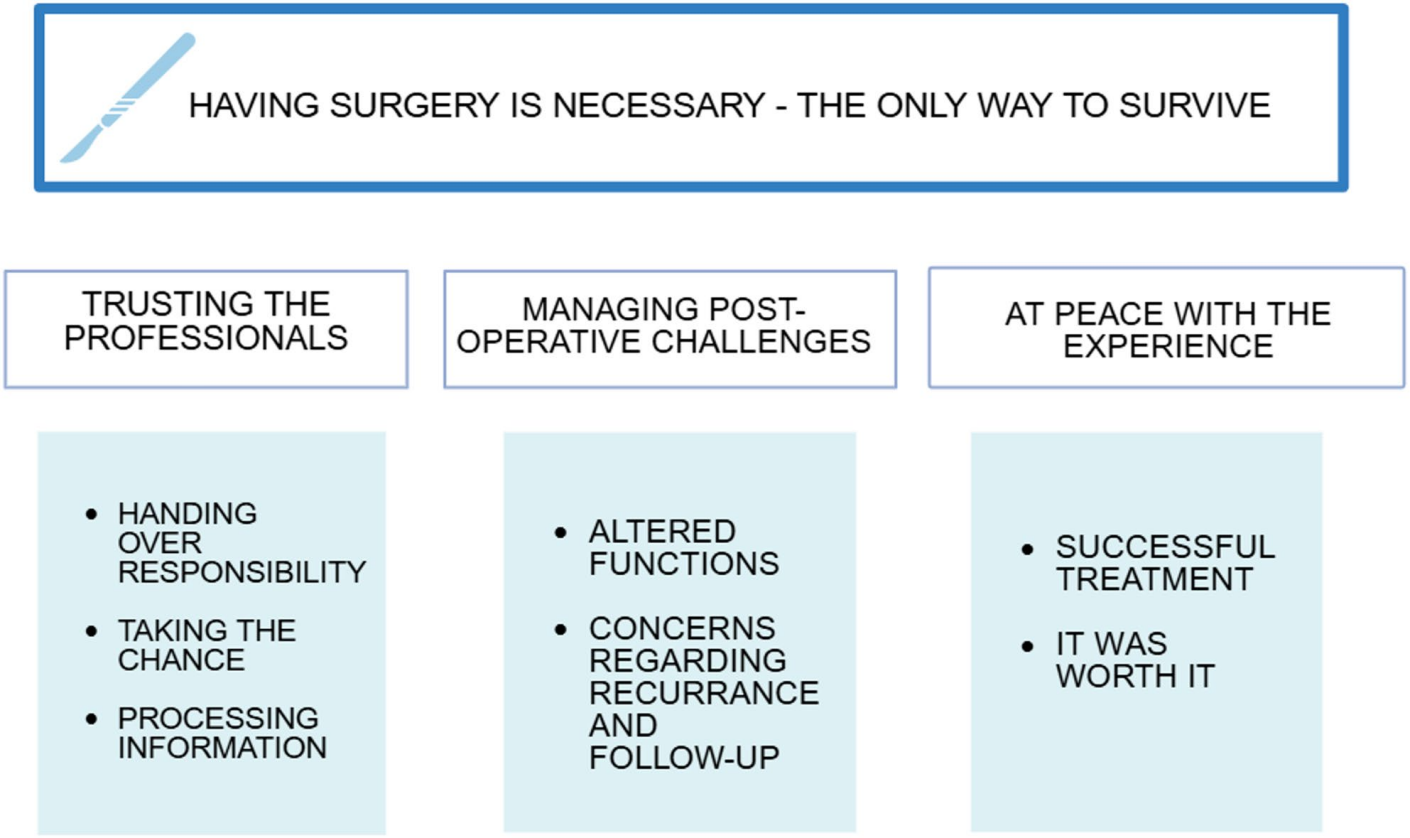
Summary of main theme, categories and subcategories

Data analysis rendered an overall theme, three main categories and seven sub-categories, displayed in Fig. 2.

### Having surgery is necessary – the only way to survive

Throughout the interviews, patients conveyed the mindset that the operation was in fact the only alternative if they were to survive. As a part of this, patients described a trust in the healthcare system, different ways of managing post-operative challenges as well as being at peace and content with the experience.

### Trusting the professionals

Participants expressed significant trust in the healthcare professionals they met through the treatment process, from diagnosis to the post-operative period. There was a strong will to let the physicians make the medical decisions and in a clear way deliver a treatment plan. Having surgery, regardless of risks, was thought of as the chance to survive. The ability to process pre-operative information varied among the patients, in some cases the information was reassuring whereas in other cases seen as redundant.

### Handing over responsibility

With the exception of clearly communicating the wish to go through surgery, participants expressed that they did not have enough knowledge or experience to be involved in medical decision-making.

*“I do not have the knowledge or capacity to decide*,* but I did say what I wanted. I do not think I would have been operated if I had not made my opinion clear*,* that I wanted to have surgery” (ID 16)*

*“My attitude is that the healthcare should decide and then we will see what happens…”*,* (ID 4)*

*“If there would have been something [wrong] I guess they would have advised me not to have surgery” (ID 7)*

### Taking the chance

Some patients described they were aware of the increased risks of having surgery at advanced age and with certain comorbidities, whereas other did not consider that aspect before proceeding to surgery.

*“Yes*,* I realise there must be a big difference [increased risks of surgery at old age]. And harder to recover*,* I believe. A person that is physically fit and young probably recovers faster” (ID 14)*

*“No*,* I did not think about that [that there may be increased risks of surgery at old age]*,* maybe I should have. I guess that there are greater risks when you are older*,* but*,* well*,* I did not think about that” (ID 9)*

*“No*,* no*,* no*,* absolutely not [increased risks of surgery at old age]” (ID 11)*

*“No*,* actually I don’t [increased risks of surgery at older age]. The thing I have had before is high blood pressure and I take three medications for that” (ID 5)*

Regardless of their degree of awareness of how frailty, advanced age or comorbidities possibly could affect risks, having the surgery done was displayed as a chance to extend life.

*“…grateful they found it and that I got a chance to be operated. I am old*,* and I am terribly grateful they bothered*,* everything was positive …. If I would have died there that would be ok. I have had a good life and everything*,* there’s no problem” (ID 11)*

*“No*,* age does not exist to me… I had no choice*,* that’s how I felt*,* I had to take away the tumour. I did not think so much*,* I just wanted it to be done…. No matter how bad my heart is*,* I had no choice. The lump that was there was not nice to have as it was” (ID 10)*

When asked about what would happen if the patients could not receive surgical treatment, the perception was that having surgery was in fact the only option and other alternatives were not considered.

*“In that case [without surgery] I would not have been alive today*,* that is clear*,* that I understand” (ID 1)*

*“I do not want to die*,* but I will take the chance. I have lived and had a good life. Rather that*,* than slowly dying from cancer”. (ID 16)*

### Processing information

Preoperative information, both verbal and written, was given but with varying response and interest from patients.

*“I got a lot of written information*,* and I read that*,* but I don’t think I really understood it.…I did not have the energy*,* I figured that what will come*,* will come” (ID 8)*

*“You had some information of how it was going to happen and that made you calmer” (ID 15)*

*“I just did as I was told, I did not think about it, it was enough information for me” (ID 6)*

### Managing post-operative challenges

A change in function was described in most cases. There was a strive towards returning to “normal”, and the degree of functional decline and persisting symptoms varied. Having a structured follow-up was associated with a sense of security and having that reassurance was described as a way to set thoughts of the cancer aside.

### Altered functions

Patients seemed to focus mostly on having the surgery done, but in a lesser extent on post-operative function and challenges. Among patients who received an ostomy, especially in those cases where they had not learned to independently manage it, the increased dependence on others and insecurity regarding how to handle problems, were described as limitations in daily life.

*“I find it hard to go visit people*,* going to concerts etc.*,* because I am afraid there will be leakage. That has happened several times and it is not fun. That limits me” (ID 3)*

*“There were some accidents in the beginning*,* but as soon as I learned to manage the ostomy myself it has been working perfectly” (ID 9)*

*“If I did not have the ostomy*,* I would probably have a more normal life … I cannot live my life as I used to” (ID 8)*

*“What if there is leakage from the ostomy [when leaving the house] and I cannot manage it by myself” (ID 7)*

Other examples of impaired function, that has affected the patient’s way of life, was altered bowel function and decreased physical activity and energy.

*“I understood that the surgery was necessary. But I did not think it could be this bad afterwards. I thought ´just do the surgery and have it done with´” (ID 8)*

*“I walk slowly*,* and I cannot make it to the bathroom in time. I do not like that. It is so much work cleaning the floors.” (ID 11)*

*“I keep thinking about going out for walks*,* but that has become less. I do not walk very much” (ID 2)*

### Concerns regarding recurrence and follow-up

Having a planned follow-up was reassuring and seen as an integral part of a successful treatment. Consequently, patients who were not planned for radiologic follow-up described this as a cause of worry and did not always understand the rationale behind the decision.

*“I am planned for a check-up in September*,* up until then I do not need to worry*,* right? I trust that they would have called me in sooner if there was anything to worry about.” (ID 16)*

*” I felt like they ditched me [when radiologic follow-up was not planned]*,* I think everyone should have the right to good treatment*,* age should not matter that much” (ID 3)*

### At peace with the experience

In alignment with seeing the procedure as a chance to survive, participants did not describe any regrets. However, there were differences in their experiences throughout the treatment period. Despite persistent challenges, the patients expressed a high level of acceptance.

### Successful treatment

Patients were asked to describe what factors were important to them to assess if the treatment was successful or not. Having a radical surgery, without recurrences, was in many cases the obvious answer to this. Others also emphasised the importance of maintained physical and cognitive function as a necessity to deem the treatment as successful.

*“To get back to the place I was before*,* preferably in a bit better shape. I assume that if you do not get rid of the cancer*,* then you die. And the best chance to be able to live as before*,* is to get rid of the cancer*,* that’s the main thing as I see it” (ID 16)*

*“For me [a successful treatment]*,* it is to come back and be as a ‘normal person’*,* without impairments. Being able to move around*,* go outside*,* and still function with thoughts and such” (ID 2)*

*“Well… that must be to be able to live as I do now*,* without pains or other such things [a successful treatment]. As long as it is like that I am pleased” (ID 9)*

*“That I am alive*,* of course [definition of successful treatment]. I have been given an extra year*,* so far” (ID 10)*

### It was worth it

Patients were asked to consider if they believe it was worth going through the process of surgery and possible other treatments, and if they would choose to do it again, should they be diagnosed with another tumour.

*“It is a big thing*,* and it is in the way*,* that limits me [abdominal hernia]… It was worth it*,* if I had not done it*,* I would have died*,* so in that way it is worth it” (ID 10)*

*“Yes*,* absolutely [do it again]! No*,* there is nothing to hesitate about. I am so pleased with the care I have been given*,* there is nothing to complain about*,* nothing” (ID 5)*

*“Yes*,* it was not as difficult as I had imagined beforehand*,* when I did not know anything. I am positively surprised” (ID 9)*

## Discussion

The most evident opinion among participants in this study was that there was never another option than having surgery, despite other complicating factors such as advanced age, frailty and comorbidities.

In most cases there is a clear recommendation to proceed with surgery, but there are situations where the clinician might want to have a discussion with the patient regarding which option (surgery or best supportive care) would benefit the patient most. Especially if there is a short life-expectancy, a locally advanced tumour, or other complicating factors that might favour non-operative options. It is not known to us if the option of not proceeding to surgery was discussed in any of these cases, or to what extent, nor are the medical considerations determining that within the scope of this article. However, our findings indicate that, from a patient perspective among the participants in this study, where the general opinion seems to be that surgery is the only alternative, a discussion regarding refraining from surgery may be difficult. Additionally, apart from wanting to state the wish of having surgery, the participants did not express a will to be involved in further medical decision-making. Rather, they conveyed the opinion that the professionals should decide what would be best for them. These results are in line with previous work of Sokas et al. [25] who interviewed older adults three months after having an emergency surgery. The participants views were that there was no other option than proceeding with surgery, despite other complicating factors. The surgery was described as a matter of life and death, and aspects of possible future impairments were not incorporated in the decision. Interestingly, having gone through an acute illness and emergency surgery did not seem to increase the incentive of being involved in future health-related decision making [25].

All participants in this study confirmed they had been given both written and verbal information regarding the diagnosis and planned treatment. However, there was a wide variety of how the information was received. There were participants describing that the information was reassuring and helpful whereas others described it as redundant or even uninteresting. Health literacy is defined as a persons’ ability to gain, process and use health information and in general it decreases with advancing age [26]. Further, health literacy is related to an individuals’ educational level [27]. It can be assumed that individuals with low levels were present also in our population. However, since we lack information on the educational status of all participants, we cannot draw any conclusions about the significance of education in these older patients’ ability to understand and use the information provided. Furthermore, additional unknown factors, such as fatigue, cognitive function, and emotional state, may also have influenced participants’ ability to comprehend and process the information. To enable implementation of person-centred care where patient empowerment is a key concept, health care professionals need to explore patients’ pre-understanding, current health status, and preference for involvement, and adjust the level and degree of information accordingly. This may, for example, involve the use of established communication approaches shown to support older patients, such as clear, jargon-free language, a slower pace of communication, and checking understanding through techniques such as teach-back [28].

The idea that elderly, comorbid patients tend to prioritise functional outcome, such as remaining physical function and independency, over morbidity and mortality is supported by some previous studies [29, 30]. On the other hand, there are studies pointing in the opposite direction, one study of elderly patients with advanced heart failure described that a majority of patients valued longevity more than preserved quality of life, however the tendency to favour increased survival decreased with advanced age [31]. In the present study, frail older adults diagnosed with colorectal cancer often appeared to view surgery as a definitive opportunity for cure, which may have contributed to a willingness to accept substantial risks.

Some of the patients in our study experienced decreased functional levels and had not recovered to the extent that they were able to resume their previous activities. Frailty is associated with increased vulnerability, and the risk of functional decline in connection with surgical treatment may be considerable. Exercise and nutrition are recommended interventions [32]. However, rehabilitation in the colorectal surgical context has largely focused on the early postoperative phase to support timely discharge. In our clinical setting, structured rehabilitation was limited to the initial early mobilization strategy embedded within the standard ERAS protocol and few of our subjects had initiated any rehabilitation on their own. Having in mind that the purpose of the surgery is to extend life while maintaining as much function as possible, a structured rehabilitation plan is likely to be beneficial. Future intervention studies may consider postoperative rehabilitation alongside preoperative optimisation in this high-risk group, in line with recent initiatives within the ERAS framework that are now evaluating the role of more structured rehabilitation following discharge [33].

Accepting a certain decline in several areas might be legitimate with advancing age, but too easily accepting a poor post-operative function may also be ineffective if there is a chance to improve the situation with targeted actions. That older adults can tend to avoid seeking help from health services has been described previously [34]. For health care professionals it is important to realise that frail older adults may be reluctant to make the healthcare system aware of persistent symptoms or problems following their treatment. This should be taken into consideration when structuring follow-up for these patients. In many cases patients are advised to get in touch if they experience problems, for these patients it might be more suitable with a targeted follow-up to be able to find manageable problems. Among patients in this cohort, recurrent issues primarily concerned ostomy dysfunction and altered bowel habits. These symptoms were sometimes perceived by patients as inevitable consequences of surgery rather than problems amendable to management or treatment. Structured follow-up, for example through targeted telephone follow-up focusing on these areas, may help identify patients with treatable problems and enable adequate treatment. Furthermore, educational efforts aimed at patients, relatives, and home-help service personnel may contribute to improved recognition and management of postoperative symptoms.

Another finding is the perception of what characterises a successful treatment. The opinions in this material focused on having a radical surgery: “getting rid of the tumour”, but preferably in combination with maintained physical and cognitive functions. However, even patients who were severely affected from ill functioning ostomies, altered bowel movements or fatigue still considered the surgery successful. Olsson et al. [35] described similar results in a qualitative study of older adults recovering from hospitalisation of different causes (acute illness, injury or surgery). Even if there was a strong wish to go back to “normal” and living independently, patients who experienced physical deterioration or decreased independence following hospitalisation had a high level of acceptance which was described as a way of managing the changes [35]. It is worth considering that participants in this study were mild to moderately frail (CFS 4–6) and had survived several months after treatment. This is likely to have an influence on our findings.

Several measures were taken to assure that the design of this qualitative study enabled trustworthiness [23, 36]. The purposive sampling procedure made it possible to include patients of different sex, various ages, both colon- and rectal cancer, with a spread of elapsed time since surgery and patients who received and did not receive adjuvant treatment and follow-up. Using quotations in the Results section is a way to achieve credibility as the patients’ own words are used to explain our interpretation of the meaning, thereby enhancing transparency.

Participants were informed that the interviewers did not play a part in any further treatment or follow-up, to ensure authenticity. The author group consists of researchers with specific clinical and scientific knowledge of frailty as well as in the colorectal cancer surgery field. This pre-understanding is important to acknowledge as it may influence the interpretation of patients’ narratives. To avoid letting the researchers’ pre-conceptions influence interpretation of data, textural analysis consisted of coding the sentences close to the text, leaving interpretation to the very final stage when describing overall themes. Furthermore, an iterative triangulation process was employed throughout the analysis to reach agreement within the author group and to incorporate diverse professional perspectives in the interpretations of data.

## Limitations

Despite the wide time range of interviews (6–22 months post-surgery) being a way of capturing patient experiences from different temporal perspectives, it is also a limitation. Patients who were interviewed closer to the time of surgery are more likely to recall details regarding the time surrounding the surgery and the information given preoperatively, whereas patients interviewed nearly two years from surgery may have forgotten details or reconstructed their experiences over time, thereby introducing recall bias, which should be considered when interpreting the results of this study. Bias-mitigation strategies, such as cross-referencing narratives with medical records for key clinical events or stratifying analyses according to time since surgery, were not implemented in this study. The goal of this study was to investigate the overall real-life experience of surgery and recovery following treatment; however, future research could incorporate such bias-mitigation strategies to better distinguish experiences related specifically to the treatment from broader health changes following colorectal cancer treatment, progress of comorbidities and ageing.

Furthermore, the study includes patients with mild to moderate frailty (CFS-9 scores of 4–6), and the conclusions drawn from this manuscript cannot be extrapolated to patients with more advanced degrees of frailty. It may be hypothesized that the mindset toward undergoing surgery might change with increasing frailty and consequently more limited functional capacity; however, this was beyond the scope of the present study and should be explored in future research. Most patients (88%) in this material had colon cancer, indicating that the perspectives of patients undergoing rectal cancer surgery and treatment are not fully represented. Additionally, all patients included in this material had survived surgery for at least six months. Given that frail older adults undergoing colorectal cancer surgery have an increased risk of postoperative mortality compared with non-frail patients, the perspectives of those who do not survive the postoperative period are not captured. Finally, this study was conducted in a Sweden within an ERAS framework, and the results may not be transferable to other countries or clinical settings. Consequently, the results of this study cannot be transferred to all frail older adults with colorectal cancer, particularly those with the most adverse outcomes, severe frailty and rectal cancer.

Finally, there are limitations inherent to qualitative methodology that must be acknowledged. The findings of qualitative studies should be considered exploratory and hypothesis-generating. The small sample size, while focusing on achieving depth and different perspectives, rather than generalisability, limits the possibility to apply the results of this study on an entire population.

## Conclusion

In this study of patients with mild to moderate frailty who had been diagnosed with colorectal cancer, underwent surgical treatment, and survived at least six months postoperatively, participants generally considered surgery as the only option for survival, and therefore appeared willing to accept substantial risks. Participants valued clear recommendations regarding treatment and expressed limited desire for active involvement in medical decision-making. Successful surgery was commonly understood as improving, or at least not further impairing, physical and cognitive function; however, a high level of acceptance of postoperative changes appeared to serve as a way of managing altered function. Given indications of a help-avoidance behaviour among some participants, greater responsibility may lie on the health services to proactively identify and address actionable impairments during follow-up. Despite post-operative challenges, participants in this study often regarded the treatment as worthwhile.

## Supplementary Material

Additional file 1: COREQ_Checklist MN 251003.pdf. A completed COREQ checklist.

Additional file 2: Patient information English BMC Ger.pdf. Written patient information regarding study, translated to English.

Additional file 3: Informed consent BMC Ger.pdf. Patient consent form, translated to English.

Additional file 4: Interview Guide BMC Ger.pdf. Interview guide used in the study, translated to English.

## Data Availability

Data is provided within the manuscript.

## Abbreviations

CFS: Clinical Frailty Scale
CGA: Comprehensive Geriatric Assessment
CRC: Colorectal Cancer
ERAS: Enhanced Recovery After Surgery
HRQoL: Health Related Quality of Life
MDT: Multidisciplinary Team Conference
PCC: Person–centred Care
VGR: Region Västra Götaland
QoL: Quality of Life
