# Supplementary material for: “Having surgery is necessary” – a qualitative analysis of the experiences of frail older adults treated with, and recovering from colorectal cancer surgery
Source: BMC Geriatr. 2026 Mar 17;26:484. doi: 10.1186/s12877-026-07356-3 (PMC13064332; doi:10.1186/s12877-026-07356-3)
Supplement: Supplementary file 2 — Additional file 2: Patient information English BMC Ger.pdf. Written patient information regarding study, translated to English. [file 12877_2026_7356_MOESM2_ESM.pdf]

Supplementary information to the manuscript"" Having surgery is necessary" - a qualitative analysis of the experiences of frail older adults treated with and recovering from colorectal cancer surgery". Submitted to BMC Geriatrics. Authors: Maria Normann*, Niklas Ekerstad, Mattias Prytz, Erika Björklund, Kristina Åhlund.

2024-12-09 (Translated to English 2025-10-06)

Information to research participants

”Can preoperative comprehensive assessment and care reduce mortality following surgery for colorectal cancer among frail older adults” – interview study

You have previously been offered and accepted participation in a clinical study at the department of surgery. The study evaluates the effect of an individualised assessment and care for frail older adults prior to elective surgery for colorectal cancer. Patients included in the study are randomized to control group (receiving care according to best practice) and intervention group (receiving care according to best practice with the addition of comprehensive geriatric assessment and care).

You are included in the control group of the study, and we would like to ask you if you, in addition to participating in the main study, would like to take part in an interview and describe your experiences.

Background and aim

The aim of the study is to explore ways to improve the care for frail older adults with colorectal cancer, one way of achieving this is to enhance the understanding of how these patients experience the treatment.

We are approaching you with this inquiry, asking if you are willing to participate in an interview study. If you choose to participate you will be contacted by phone to settle a time for the interview. You will be able to tell your story and describe your experiences concentrated on the period before and after the surgery. It is up to you to decide if you want the interview to take place in your home, or if you would rather like to do it at the hospital. The interview will take approximately 45 minutes and will be audio recorded.

Voluntarism

Your participation is voluntary. If you do not wish to take part in this study, it will have no consequences for you or your future contacts with the health care. If you wish to withdraw your consent of participation and terminate your participation you do so by contacting any of the named persons below.

Data management and secrecy

Your answers and the results will be treated in a way to prevent any unauthorized access. Your personal data will be handled according to GDPR. Information regarding the study will be entered in your medical records and the results will be kept in a password protected computer only available for the researchers directly involved in the study. The results will be presented at group level and will not contain any personal data. You are entitled to demand an extract from the registry once a year, regarding your personal data. You are also entitled to demand corrections or deletion of personal information. Should you wish to withdraw your consent and terminate your participation in the study that will not affect your future care. Any complaints can be conveyed to the Swedish Authority for Privacy Protection, imy@imy.se or 08-657 61 00. If you have any concerns regarding the management of your personal information or want to receive advice regarding your rights regarding this you can turn to the data protection officer in NU-Hospital group nu.dso@vgregion.se or 010-4350000. This study is approved by the Swedish Ethical Review Authority, Dnr: 2024-07171-02.

Study group

Research principal: Region Västra Götaland

Maria Normann
E-mail: maria.normann@vgregion.se
Phone: 0734-166 982

Kristina Åhlund
E-mail; kristina.ahlund@vgregion.se
Phone: 070-10 39 863

Mattias Prytz
E-mail: mattias.prytz@vgregion.se
Phone: 010-435 34 00

*Corresponding author, Department of Surgery, Institute of Clinical Sciences, Sahlgrenska Academy, University of Gothenburg, Gothenburg, Sweden. Department of Surgery, Region Västra Götaland, NU-Hospital Group, Trollhättan, Sweden, maria.normann@vgregion.se
