# Supplementary material for: “Having surgery is necessary” – a qualitative analysis of the experiences of frail older adults treated with, and recovering from colorectal cancer surgery
Source: BMC Geriatr. 2026 Mar 17;26:484. doi: 10.1186/s12877-026-07356-3 (PMC13064332; doi:10.1186/s12877-026-07356-3)
Supplement: Supplementary file 3 — Additional file 3: Informed consent BMC Ger.pdf. Patient consent form, translated to English. [file 12877_2026_7356_MOESM3_ESM.pdf]

Supplementary information to the manuscript"" Having surgery is necessary" - a qualitative analysis of the experiences of frail older adults treated with and recovering from colorectal cancer surgery". Submitted to BMC Geriatrics. Authors: Maria Normann*, Niklas Ekerstad, Mattias Prytz, Erika Björklund, Kristina Åhlund.

2024-12-09 (Translated to English 2025-10-06)

# Informed consent

## ”Can preoperative comprehensive assessment and care reduce mortality following surgery for colorectal cancer among frail older adults” – interview study

You have been invited to partake in an interview study concerning patient experiences of treatment for bowel cancer. The participation is voluntary and your consent to participate can at any time be withdrawn. Your personal data will be treated according to GDPR and in accordance with the Public Access to Information and Secrecy Act.

I have received written and verbal information regarding the study. I accept the terms and hereby approve that my coded interview transcripts can be transferred to the project database and used for research within the described project.

Place and date written by the participant

________________________________________

Date, place

________________________________________

Signature

________________________________________

Printed name and ID

I have explained the study and have received the participants consent of participation

VÄSTRA GÖTALANDSREGIONEN

Date

Signature, printed name

*Corresponding author, Department of Surgery, Institute of Clinical Sciences, Sahlgrenska Academy, University of Gothenburg, Gothenburg, Sweden. Department of Surgery, Region Västra Götaland, NU-Hospital Group, Trollhättan, Sweden, maria.normann@vgregion.se
