# Supplementary material for: “Having surgery is necessary” – a qualitative analysis of the experiences of frail older adults treated with, and recovering from colorectal cancer surgery
Source: BMC Geriatr. 2026 Mar 17;26:484. doi: 10.1186/s12877-026-07356-3 (PMC13064332; doi:10.1186/s12877-026-07356-3)
Supplement: Supplementary file 4 — Additional file 4: Interview Guide BMC Ger.pdf. Interview guide used in the study, translated to English. [file 12877_2026_7356_MOESM4_ESM.pdf]

2024-11-22
(Translated to English 2025-10-03)

Supplementary information to the manuscript”” Having surgery is necessary” - a qualitative analysis of the experiences of frail older adults treated with and recovering from colorectal cancer surgery". Submitted to BMC Geriatrics. Authors: Maria Normann*, Niklas Ekerstad, Mattias Prytz, Erika Björklund, Kristina Åhlund.

” Can pre-operative comprehensive geriatric assessment and care reduce mortality following surgery for colon- or rectal cancer among frail older adults” Interview study of patients in the control group.

The aim of this study is to explore frail older adults’ experiences of having surgery for colon- or rectal cancer. The interviews will be semi-structured and focus on the following areas: the experiences of the period prior to surgery, in relation to the surgery and following surgery. The following questions will be used as examples to highlight these areas.

Interview guide

The interviews will start with the following question:

- What were your thoughts when you were told that you were going to have surgery for your cancer?

Prior to surgery

Participation and person-centeredness

1. Do you feel that you had any possibility to influence the treatment that you were going to receive for your bowel cancer?
2. When you look back on the treatment you received, do you feel you got enough information to make the decision to proceed with surgery?
    a. Did you have information about the” right” things before the surgery, or do you feel there was lacking information?

Expectations

3. How would you describe the period from when you received notice about the planned treatment to the surgery?

After the surgery

4. How did you experience the time straight after the operation?
    a. Did you receive support from the health care system? From relatives/friends?
    b. Did you have a lot of questions?
    c. Did you feel worried?

The present – future

5. If you were to be diagnosed with a new tumour in the bowel, would you go through the same kind of treatment again?
6. What is the most important measure that the treatment was successful, according to you?
7. Has the treatment affected your ability to perform regular activities?
8. Do you feel that your physical well-being today has been affected by the cancer treatment?
    a. Do you feel that your mental well-being has been affected by the cancer treatment?
9. How has your quality of life been affected by the cancer treatment?

*Corresponding author, Department of Surgery, Institute of Clinical Sciences, Sahlgrenska Academy, University of Gothenburg, Gothenburg, Sweden. Department of Surgery, Region Västra Götaland, NU-Hospital Group, Trollhättan, Sweden
maria.normann@vgregion.se
